# Impact of SGLT2 Inhibitors on Heart Failure: From Pathophysiology to Clinical Effects

**DOI:** 10.3390/ijms22115863

**Published:** 2021-05-30

**Authors:** Giuseppe Palmiero, Arturo Cesaro, Erica Vetrano, Pia Clara Pafundi, Raffaele Galiero, Alfredo Caturano, Elisabetta Moscarella, Felice Gragnano, Teresa Salvatore, Luca Rinaldi, Paolo Calabrò, Ferdinando Carlo Sasso

**Affiliations:** 1 Department of Translational Medical Sciences, University of Campania “Luigi Vanvitelli”, I-80131 Naples, Italy; g.palmiero@hotmail.it (G.P.); arturo.cesaro@unicampania.it (A.C.); elisabetta.moscarella@unicampania.it (E.M.); felice.gragnano@unicampania.it (F.G.); paolo.calabro@unicampania.it (P.C.); 2 Division of Cardiology, A.O.R.N. “Sant’Anna & San Sebastiano”, I-81100 Caserta, Italy; 3 Department of Advanced Medical and Surgical Sciences, University of Campania Luigi Vanvitelli, I-80138 Naples, Italy; erica.vetrano@unicampania.it (E.V.); piaclara.pafundi@unicampania.it (P.C.P.); raffaele.galiero@unicampania.it (R.G.); alfredo.caturano@unicampania.it (A.C.); luca.rinaldi@unicampania.it (L.R.); 4 Department of Precision Medicine, University of Campania Luigi Vanvitelli, I-80138 Naples, Italy; teresa.salvatore@unicampania.it

**Keywords:** SGLT2 inhibitors, heart failure, pathophysiology, type 2 diabetes, cardiovascular risk

## Abstract

Heart failure (HF) affects up to over 20% of patients with type 2 diabetes (T2DM), even more in the elderly. Although, in T2DM, both hyperglycemia and the proinflammatory status induced by insulin resistance are crucial in cardiac function impairment, SGLT2i cardioprotective mechanisms against HF are several. In particular, these beneficial effects seem attributable to the significant reduction of intracellular sodium levels, well-known to exert a cardioprotective role in the prevention of oxidative stress and consequent cardiomyocyte death. From a molecular perspective, patients’ exposure to gliflozins’ treatment mimics nutrient and oxygen deprivation, with consequent autophagy stimulation. This allows to maintain the cellular homeostasis through different degradative pathways. Thus, since their introduction in the clinical practice, the hypotheses on SGLT2i mechanisms of action have changed: from simple glycosuric drugs, with consequent glucose lowering, erythropoiesis enhancing and ketogenesis stimulating, to intracellular sodium-lowering molecules. This provides their consequent cardioprotective effect, which justifies its significant reduction in CV events, especially in populations at higher risk. Finally, the updated clinical evidence of SGLT2i benefits on HF was summarized. Thus, this review aimed to analyze the cardioprotective mechanisms of sodium glucose transporter 2 inhibitors (SGLT2i) in patients with HF, as well as their clinical impact on cardiovascular events.

## 1. Heart Failure Epidemiology in Type 2 Diabetes

Type 2 diabetes mellitus (T2DM) is a systemic, complex, chronic disease. Its prevalence has been growing over the last decades, mostly due to increased obesity and sedentary life. T2DM is burdened by an elevated risk for several cardiovascular diseases (CVDs), with heart failure (HF) as a more common initial presentation rather than myocardial infarction (MI) [1]. HF estimated prevalence among T2DM patients ranges between 9–22%, even higher in those aged ≥60 years old [2,3,4,5]. The risk of HF in T2DM patients, almost double that in the general population, is affected by several risk factors [6]. Particularly, a longer disease duration, obesity, hypertension, coronary artery disease (CAD), peripheral arterial disease (PAD), nephropathy, retinopathy and higher NT-proBNP increase the risk of HF in T2DM [7,8,9]. Moreover, in the Framingham study, a gender difference was reported, with a five-fold and 2.4-fold higher risk in diabetic women and men, respectively [10]. HF patients are often insulin-resistant, which can, in turn, either boost diabetes onset or worsen it [11]. In fact, several large cohort studies have reported an incidence of diabetes in patients affected by HF ranging between 30% and 50%, also suggesting a mutual relationship between these two diseases [12,13]. HF and T2DM, when coexistent, are associated with a worse morbidity and mortality outcome, and T2DM further represents a predictor of symptomatic HF [14].

HF hospitalization for acute episodes of decompensation represents a major human and economic burden [15], and it is affected by both gender [16] and frailty [17].

In 2008, the US Food and Drug administration issued pharmaceutical industries to assess the cardiovascular outcome of the antidiabetic therapy, beyond glycemic control [18]. Up until gliflozins commercialization and evidence from randomized control trials, no antidiabetic therapy showed significant improvements in HF hospitalization [19]. Moreover, while, initially, this benefit was attributed to the diuretic and antihypertensive effect of this class of drug, mechanisms other than SGLT2 inhibition have been recently proposed.

Thus, aim of this review is to assess the cardioprotective role of sodium glucose transporter 2 inhibitors (SGLT2i) in patients affected by both these conditions.

## 2. Heart Failure Pathophysiology in Type 2 Diabetes

T2DM owns multiple contributing mechanisms in the development of myocardial dysfunction, which affect cardiac relaxation, contractility and compliance. Structural heart disease in T2DM may develop either from myocardial ischemia or infarction, mostly due to increased atherosclerosis, atherogenic dyslipidemia and endothelial dysfunction. All of these may, in turn, lead to thrombosis, inflammation and vulnerable coronary plaque [20]. Diabetic cardiomyopathy, indeed, is defined as the presence of either diastolic or systolic cardiac dysfunction in patients with diabetes in the absence of other causes of cardiomyopathies. The term was first coined in 1972 by Rudler et al. after the post-mortem evidence of cardiomegaly in the absence of major CAD in T2DM patients [21].

Hyperglycemia plays a key role in HF development in T2DM patients, as revealed by both clinical and preclinical investigations [22,23]. In fact, a rise in HF onset by 8–36% for each 1% increase in glycosylated hemoglobin (HbA1c) has been reported [5,24,25,26]. The underlying mechanism is mainly regulated by local renin–angiotensin–aldosterone system (RAAS) activation, with an increase in the angiotensin II and aldosterone levels. These, in turn, induce cardiac hypertrophy and fibrosis and worsen diastolic dysfunction [27]. Moreover, high glucose levels are responsible for the formation of nonenzymatic advanced glycation end products (AGEs) and glucose metabolites such as b-N-acetylglucosamine, which can negatively affect cardiac contractility and relaxation by targeting Ca2þ/calmodulin-dependent protein kinase II, phospholamban and the myofilaments [28,29]. These products modify multiple mitochondrial proteins, thus also affecting the mitochondrial metabolic function [30,31,32]. In addition, the hyperglycemia degree is also associated with increased HF hospitalization, death and diastolic dysfunction, which may be improved with a better glycemic control [33,34,35,36,37,38,39,40].

Cardiac and noncardiac T2DM complications have been related to oxidative stress, increased in TD2M and mostly due to several abnormalities (e.g., hyperglycemia, inflammation and dyslipidemia) [41,42,43]. In fact, oxidative stress leads to impaired cardioprotective downstream pathways, thus resulting in cardiomyocyte calcium handling alterations, reduced cardiac contractility and relaxation [35,44]. The inflammatory signal has been further suggested as a key pathophysiological mechanism of myocardial dysfunction, as also suggested in HF of other etiologies, and it is mostly upregulated due to epicardial fat hypertrophy [22,45,46,47]. As a consequence, an increase in leptin production, a promoter of myocardial inflammation, triggers an impairment of paracrine adipokines regulation [46]. Moreover, the inflammatory state also boosts oxidative stress by increasing the leukocyte migration and elicits endothelial reactive oxygen species (ROS) production through the activation of nicotinamide adenine dinucleotide phosphate oxidases, thus affecting the coronary microvascular [48]. A high ROS level occurs in cardiomyocyte autophagy, apoptosis or necrosis and is able to decrease NO bioavailability due to NO diversion to peroxynitrite by superoxide anion, which leads to vasodilator impairment. Consequently, left ventricular diastolic dysfunction is facilitated [46,48]. Inflammation has also been implied in a recently recognized pathologic process named endothelial-to-mesenchymal transition (endoMT). It seems that both TGF-β and the Slug signaling pathways are implied in the endoMT endothelial cell shift toward mesenchymal cells, thus leading to fibrosis and ventricular remodeling [49].

Heart ATP production mostly derives from the oxidation of fatty acids and carbohydrates. However, in T2DM, high free fatty acid (FFAs) levels and impaired glucose uptake due to insulin resistance (IR) result in a reduction of glucose oxidation. The shift in fatty acid metabolism is less efficient due to an increased myocardial oxygen demand, lipotoxicity from increased free oxygen radical production and sarcoplasmic reticulum calcium uptake impairment. This modification in cell energy metabolism further depletes the cardiomyocytes of fuel, thus boosting diastolic dysfunction development [50,51]. The cell energy metabolism is also worsened by autonomic dysfunction, common in T2DM, and by perfusion abnormalities. Autonomic dysfunction can rise earlier than expected in T2DM patients and affects vascular function, especially the coronary one [52]. A higher sympathetic activity results in an increase in the oxygen demand, also boosted by a fatty acid metabolism shift, thus leading to cardiac remodeling and promoting HF development [53,54]. Moreover, beyond macrovascular disease and an increase in the arterial load, T2DM is also burdened by a microvascular perfusion impairment due to structural changes, abnormal nitric oxide metabolism, endothelial dysfunction and coronary atherosclerosis. This limits the coronary vasodilatation and rarefaction and promotes cardiac fibrosis and diastolic dysfunction [55]. Moreover, the opioid system, which seems related to IR [56], also plays an important role in HF [57].

**Figure 1:**
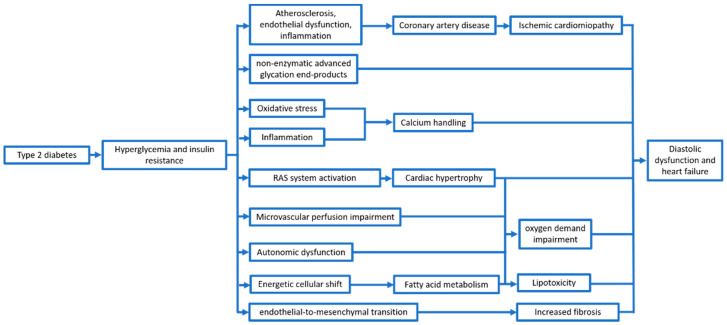
Main mechanisms leading to ventricular dysfunction in type 2 diabetes patients.

Therefore, beyond the wearing out and the reduction of global cardiac function, T2DM and, particularly, hyperglycemia and the proinflammatory status induced by IR, seem to be a trigger for several mechanisms (Figure 1).

## 3. SGLT2 Inhibitors Cardioprotective Mechanisms

**Figure 2:**
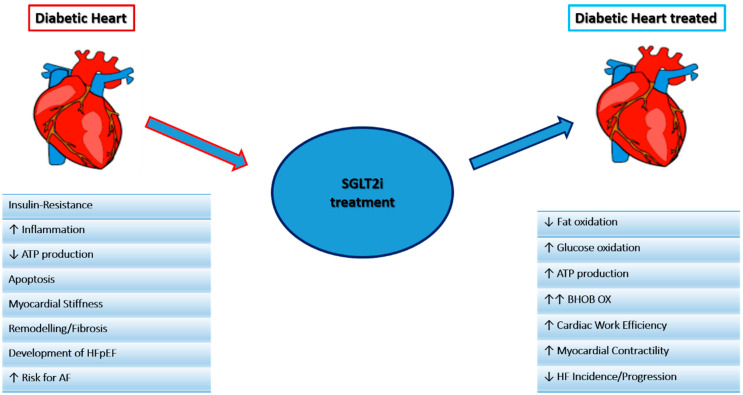
Main mechanisms of the SGLT2i effect on the heart.

**Figure 3:**
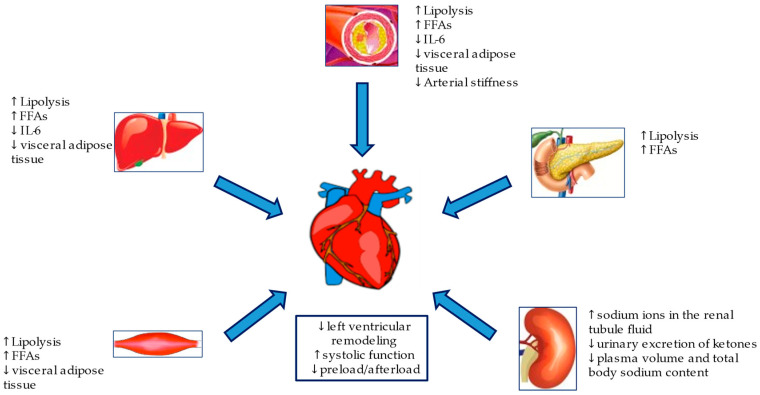
Metabolic and hemodynamic protection mechanisms of SGLT2i.

SGLT2i, either known as gliflozins, represent an effective and innovative treatment option for patients with T2DM. This class of drugs, beyond the simple glycemic control, has also been demonstrated effective in the management of medium to long-term DM2-related complications. SGLT2i have also demonstrated a significant reduction in atherosclerosis-related events, hospitalizations for HF and cardiovascular and all-cause mortality [58,59,60,61], achieved by several mechanisms hereinafter discussed. The mechanisms are summarized in Figure 2 and Figure 3.

### 3.1. Metabolic Mechanisms

A healthy heart needs a large amount of energy to keep a normal contractile function, as it consumes several substrates, including glucose and FFAs, with over 95% of ATP provided by mitochondrial oxidative phosphorylation and, to a lesser extent, by glycolysis [62]. Under stressful conditions (e.g., HF and/or T2DM), glucose used by the heart muscle is compromised, thus entrusting most of the metabolism to FFA consumption, less efficient due to an increased demand for oxygen by the myocardium. Moreover, a lipotoxicity due to a higher production of reactive oxygen species (ROS) and impaired absorption of calcium from the sarcoplasmic reticulum may occur, with the subsequent development of diastolic dysfunction [51,63].

Ketone bodies represent a good alternative substrate, able to improve the cardiac metabolic efficiency. Some studies on humans and animal models have shown an improvement of the cardiac function and metabolism by beta-hydroxybutyrate (β-OHB), thus inducing reverse ventricular remodeling, with a consequent improvement in the cardiac output and diastolic function [64,65]. Moreover, the ketone bodies also exert an anti-inflammatory role by suppressing the activation of P3 receptor inflammasome activity (NLRP3), similar to the nucleotide oligomerization domain [66].

SGLT2i are well-known to own intrinsic metabolic mechanisms, which might associate with the development of euglycemic ketoacidosis. Particularly, SGLT2 inhibition stimulates lipolysis, with a consequent increase in FFAs. This contributes to ketogenesis and also determines higher concentrations of sodium ions in the renal tubule fluid, with a subsequent increase in positive electrical charges in the tubular lumen, in turn leading to the reduced urinary excretion of ketones, negatively charged [67]. A further contribution to ketogenesis also results from an increase in glucagon levels, increased as an effect secondary to the reduced insulin excretion or to alpha-pancreatic cells activity, expressing SGLT2 [68]. Such metabolic changes have been postulated to offer a potentially good option to improve cardiac efficiency, thus preventing HF [64,69]. Hence, SGLT2i, by decreasing the glucose plasma levels, trigger an increase of circulating ketones [70]. The beneficial effects of the increased ketogenesis due to SGLT2i were observed in a nondiabetic pig model of HF, where Empagliflozin improved the left ventricular remodeling and systolic function by improving the cardiac energy [71]. Some authors also suggested the promotion of the breakdown of branched-chain amino acids (BCAAs), boosted by SGLT2i, as an alternative fuel source [72].

Both sodium and calcium homeostasis in the myocardium are critical to guarantee an efficient excitation–contraction coupling [73]. The balance of these two electrolytes is compromised in HF. There is an increase in the myocyte intracellular sodium levels, which results in a higher activity of the Na^+^/Ca^2+^ exchanger and a subsequent increase in the calcium levels in the sarcoplasmic reticulum. Although improving the cardiac contractility and cardiac dysfunction, this mechanism is also burdened by an increased risk of arrhythmia and oxidative stress due to reduced mitochondrial calcium levels induced by the mitochondrial activation of the Na^+^/Ca^2+^ exchanger [74,75]. Therefore, increased sodium and myocardial calcium levels may represent a factor involved in cardiovascular death and HF [74,76].

In diabetic individuals, the increased sodium intracellular concentration is exposed to a much higher risk [75]. In this context, the sodium–hydrogen exchanger 1 (NHE1) and SGLT1 result upregulated, with a consequent significant increase in the intra-cytosolic sodium content [77]. Empagliflozin demonstrated lower cardiac intracellular Na^+^ and Ca^2+^, with a higher concentration of mitochondrial Ca^2+^ in rabbits and rats. Such an effect is mediated by the inhibition of NHE1 [78]. Further studies on Dapagliflozin and Canagliflozin in mice proved NHE1 inhibition as a class effect [79]. SGLT2 receptors, not expressed in the heart, better render how NHE1 inhibition is performed. In addition, the inhibition of NHE and subsequent lowering of cardiac cytosolic Na+ seems a potential class effect of SGLT2i to face Hf [80]. NHE1 and NHE3 inhibition may represent a good therapeutic tool to prevent cardiac remodeling and HF [81]. Of note, although the benefits of NHE1 inhibition have been largely demonstrated in experimental models, several studies with NHE-1 inhibitors have not obtained positive results [82]. Anyway, all these observations suggest a key rule for ionic homeostasis in the cardioprotective effects of gliflozins.

SGLT2i induce a reduction in serum uric acid (UA) levels, an important independent risk factor for HF. Hyperuricemia has also been reported with HF at the preserved ejection fraction (HFpEF) in hypertensive individuals as a predictor of the incidence involved in the pathophysiological cascade of HFpEF through various proposed mechanisms [82,83].

Another important risk factor for T2DM is dyslipidemia, which affects more than 50% of diabetic patients [84]. Adipokines, such as leptin and adiponectin, are well-known essential cytokines involved in the regulation of food intake and energy homeostasis. Adipokines are altered in obesity, IR and T2DM, thus favoring a proinflammatory state. Particularly, leptin seems involved in various cardiovascular diseases that are obesity-related, whilst adiponectin seems to exert a cardioprotective role [85,86]. Alterations of the structure and leptin metabolism determine an accumulation of epicardial fat. This results in a crucial role in HF development due to cardiac fibrosis and inflammation, thus contributing to ventricular remodeling [87]. SGLT2 inhibition reduces serum leptin and increases adiponectin concentrations, likely offering cardioprotection [88]. In a recent study, Canagliflozin was demonstrated to induce a reduction in the serum leptin levels as compared to glimepiride. A reduction in proinflammatory cytokine interleukin 6 (IL-6) was also observed, while tumor necrosis factor α (TNF-α) was not affected [89]. In another study, indeed, Dapagliflozin was reported to reduce ectopic epicardial fat, TNFα and plasminogen activator inhibitor-1 (PAI-1) [90]. This observation suggests a possible role for SGLT2i in many mechanisms involved in CV inflammation [91,92,93,94]. However, these effects may reflect changes secondary to the systemic effects of SGLT2i in therapy, including weight loss and lipolysis. The SGLT2i treatment has produced modest changes in the lipid structure, with a consequent decrease in HDL cholesterol and triglycerides levels and a 20–30% reduction in small dense LDL particles [95,96,97]. Moreover, SGLT2i was demonstrated to affect the body weight in T2DM patients, with a dose-dependent reduction between 1.6 and 2.8 kg as compared to the placebo [98]. However, the CANTATA-SU study demonstrated that the weight loss observed during the treatment with Canagliflozin was of the same magnitude at both the lowest and highest dosages (100 and 300 mg per day, respectively). Such a finding suggests that, after achieving the lower limits of the SGLT2i clinical efficacy, the initial weight lowering begins to slow down until a plateau phase [99]. Despite the persistent glycosuric effects, SGLT2i induces only modest and nonpersistent effects on the body weight, thus suggesting that those metabolic changes themselves may not represent the key reason for cardio protection in patients with HF [100]. As already known, hyperinsulinemia and excessive fat mass are predisposing factors for NAFLD development [101]. Gliflozins positively affects nonalcoholic liver steatosis, as observed evaluating the reduction of the liver fat content by magnetic resonance and Fibroscan and monitoring liver biomarkers over time in individuals with T2DM [102]. While SGLT2i affects the body composition, mainly reducing the visceral adipose tissue, this metabolic effect could further improve the cardiometabolic risk profile in T2DM patients [103]. Strong evidence supports the association between NAFLD and increased risk of CVDs, with CVDs accounting for most deaths in patients diagnosed with NAFLD [104]. Therefore, the effects of this class of drugs seem synergistically both hepato- and cardio-protective and may improve the cardiometabolic risk profile of diabetic patients [105,106].

### 3.2. Hemodynamic Protection Mechanisms

Obesity and T2DM may also lead to HFpEF, induced by an increase in cardiac preload, which develops due to the volume overload in response to plasma volume expansion. In these patients, IR and proinflammatory cytokines released by hypertrophic visceral adipocytes cause arterial stiffness, endothelial dysfunction in the arterioles and a reduction in capillary density at the systemic level and of the heart, thus increasing the cardiac afterload [107,108]. The direct cardiac effects of SGLT2i include an improvement both of the preload, secondary to natriuresis and osmotic diuresis, and of the afterload, secondary to the reduction of sodium and circulating volume, through the reduction of both systolic and diastolic blood pressure (3–5 mmHg and 2 to 3 mmHg, respectively) [109], without any increase in the heart rate and reducing the arterial stiffness [110,111].

Several mechanisms seem involved in the reduction of blood pressure (BP) induced by SGLT2i, such as a reduction in sodium reabsorption in the proximal renal tubule with a consequent increase in diuresis, the improvement of vascular function in terms of improvement in stiffness and vascular resistance and bodyweight reduction [112].

The EMPAREG-OUTCOME data first assessed the role of SGLT2i as BP-lowering drugs. This finding has been subsequently confirmed by two meta-analyses, which showed a reduction in both SBP and DBP of 2.46 mmHg and 1.46 mmHg, respectively, and in 24-h SBP and DBP of 3.76 mmHg and 1.83 mmHg, respectively [113,114,115]. Indirect data from a meta-analysis demonstrated a better efficacy of Canagliflozin 300 mg in lowering SBP than other SGLT2i, whilst no difference was observed as for DBP [98]. SGLT2i has also been found as more effective during the night than in the daytime [116], with the particular benefit to using Empagliflozin in high-risk Asian patients with uncontrolled nocturnal hypertension [110]. Moreover, the pulse wave velocity (PWV), an arterial stiffness index, decreased after 48 h of Dapagliflozin administration in a small cohort of patients with T2DM [117]. As well, the beneficial effect of Dapagliflozin on the endothelial function have been further stressed [118]. Improvement of the endothelial function was also evaluated in another study on Dapagliflozin and in vitro models [119,120]. Other arterial stiffness indicators such as central systolic BP and forward and backward pulse wave amplitude have been studied with both Empagliflozin and Dapagliflozin, with positive results [121,122].

According to a double-blind, randomized study, the sodium levels in the tissue of diabetic patients decrease after six weeks of treatment with Dapagliflozin [123]. By inhibiting SGLT2 in the proximal renal tube, gliflozins induce natriuresis [124] and glucosuria [125]. The presence of non-reabsorbed glucose and sodium in the tubule stimulates osmotic diuresis. Consequently, both the sodium and chloride concentrations are lower in the tubular fluid, thus inhibiting the NA-K-2Cl cotransporter and preventing sodium reabsorption in the loop of Henle [126,127]. As a result, there is a reduction in the plasma volume and total body sodium content [128], with consequent alterations of the cardiac preload conditions, positively affecting the left ventricular Franklin-Starling curve [129,130]. However, other diuretics, such as thiazides and loop diuretics, did not benefit the CV outcome, as reported in two studies comparing Dapagliflozin vs. hydrochlorothiazide and bumetanide, respectively [125,131]. SGLT2i may also affect the afterload by acting both on BP and arterial stiffness. According to recent studies, an increase in hematocrit associated with SGLT2 is partially attributable to the hemoconcentration due to the reduction in the volume of extracellular fluids and the improvement in diuresis [130].

The natriuretic and diuretic effects of Dapagliflozin lead to a 7% decrease in the plasma volume in individuals with T2DM, followed by a 24-h reduction in BP and a 2.2% increase in hematocrit at week 12. On the other hand, the treatment with Dapagliflozin also increased the erythropoietin (EPO) concentration and reticulocyte counts, thus increasing the hematocrit and hemoglobin values [131]. Moreover, the increase in erythropoietin has favorable effects on the mitochondrial function of cardiomyocytes, cell proliferation, inflammation and angiogenesis. It also triggers myocardium protection by increasing the hematocrit, with a consequent increase of the oxygen supply to the tissues [132]. Besides, the observed relationship between increased hemoglobin and hematocrit levels and cardioprotection after the administration of Empagliflozin (shown in the exploratory analysis of the EMPA-REG study) was associated with a decrease in the CV mortality rate [133]. However, most recent studies suggest it is more compelling to attribute the cardioprotective role of SGLT2i to the cumulative effects of both the metabolic and hemodynamic mechanisms.

### 3.3. Antiapoptotic and Antifibrosis Effects

The cardiac damage induced by hyperglycemia is also determined by increased ROS, inflammation and apoptosis [134]. High glucose levels are responsible for the formation non-enzymatic glycation end products of proteins, lipids and nucleic acids, thus inducing inflammation and consequent apoptosis and fibrosis [31]. Epicardial fat hypertrophy induces a shift in adipokines production, with a subsequent increase in leptin levels rather than adiponectin. Leptin favors the production of proinflammatory cytokines and, consequently, the expression of inducible NOS in cardiomyocytes following the activation of the nuclear factor-kappa B (NF-κB) [135]. Studies in mice have shown that SGLT2 inhibition reduces the circulating levels of chemokine 2, IL-6 and TNF-α [136]. Similar findings were also observed as for NF-κB and IL-6 in the renal tissues of diabetic mice and IL-6 and C-reactive protein (CRP) in hepatic cells and adipocytes of obese mice subjected to a diet [137,138]. Thus, SGLT2i might change the inflammatory responses in several cells of both the kidneys and other tissues by various molecular pathways, affecting the oxidative stress, hemodynamics, hyperglycemia-induced cytokine production, RAAS activation, system function immune system and obesity-related inflammation [139]. The molecular mechanisms of HFpEF progression have recently been investigated [136]. The overexpression of nitric oxide synthase (iNOS) induces a reduction in the activities of two proteins: an isoform of the binding protein X-box 1 (XBP1) and the enzyme 1α. A reduction in XBP1 expression inhibits the protein response and leads to the myocardial accumulation of destabilized proteins and increased cardiomyocyte apoptosis [140]. Schiattarella et al. showed that either a deficiency in the expression of iNOS or overexpression of XBP1 in the mice affected by HFpEF improves the phenotype, thanks to the left ventricular filling pressures and a lower reduction of pulmonary congestion. SGLT2i may inhibit iNOS expression and activate eNOS, thus causing an increased XBP1s expression and increased titin phosphorylation in heart muscle [141].

Myocardial fibrosis represents a crucial part of the cardiac remodeling, leading to HF. Lee et al. demonstrated that Dapagliflozin administration exerts a significant cardiac antifibrotic effect in the rat models of post-myocardial infarction, reducing collagen synthesis by stimulating M2 macrophages and inhibiting myofibroblast differentiation [142]. Moreover, Kang et al. demonstrated that Empagliflozin suppresses the profibrotic markers (e.g., type I collagen, a-smooth muscle actin, connective tissue growth factor and matrix metalloproteinase 2) and mitigates transcription growth factor β1 (TGF-β1)-induced fibroblast activation [143]. Another target of SGLT2i is AMP-activated protein kinase (AMPK), an enzyme acting as a regulator of metabolic homeostasis, promoting the catabolism and inhibition of anabolism [144]. As a mediator of several signaling hormones, AMPK exerts protective effects on the mitochondria and reduces inflammation, apoptosis and fibrosis. Empagliflozin has been reported to activate AMPK/Drp1 signaling in streptozotocin-induced diabetes (STZ) in mice, thus showing mitochondrial protection by the reduction of mitochondrial fixation and oxidative damage. These are followed by an improvement of the barrier function of the vascular system by the phosphorylation of nitric oxide endothelial synthase (eNOS) and reduction of microvascular endothelial lesions in coronary endothelial cells [145]. Moreover, in a model of myocardial injury from ischemia–reperfusion (I/R) and hypoxia/reoxygenation (H/R) in isolated cardiomyocytes, Empagliflozin reduced the infarcted area and improved the myocardial contractility, which affects the AMPK signaling pathways [146]. TGF-β/Smad, strongly involved in tissue fibrosis regulations, represents another signaling pathway affected by Empagliflozin, which induces its block, and a subsequent decrease in the fibrotic transformation of myocardial tissue [147]. Both animal and clinical studies have demonstrated a sympathetic inhibitory effect that, beyond being associated with the reduction of fibrosis, by itself an important arrhythmogenic substrate, suggested the role of SGLT2i in the prevention of any arrhythmic event [148]. However, currently, no study has investigated the association between the risk of arrhythmias and the use of SGLT2i in diabetic patients. Both systemic and coronary autonomic nervous system (ANS) dysfunction, potentially present in diabetic patients even in the early stages, may be modulated by SGLT2i, as demonstrated by both in vitro and in vivo studies [149]. SGLT2i causes a reduction in the ANS activity by decreasing the insulin, leptin and glucose blood levels, improving IR, hyperinsulinemia and anemia. All those contribute to a reduction of the carotid body (CB) activation, as well as of the volume of sodium and the level of protein-bound uremic toxins, which inhibit the activation of the organum vasculosum of the lamina terminalis (OVLT) in the region of the third anteroventral ventricle (AV3V) of the hypothalamus [150]. Overall, these findings suggest a potential effect of SGLT2i on inverse cardiac remodeling in HF patients with both preserved (HFpEF) and reduced (HFrEF) and, also, in nondiabetic patients. Moreover, due to this effect, SLGT2i may represent a potential new class drug for HFpEF, a setting in which many other promising drugs have failed.

### 3.4. Anti-Effects on Autophagy and Stress

Autophagy is the process by which cellular homeostasis balance is maintained after the elimination of potentially dangerous substances and the recycling of cellular components as an adaptive response to metabolic stress, including hypoxia [151]. Autophagy induction pathways involve the activation of the protein kinase activated by adenosine monophosphate (AMPK), sirtuin-1 (SIRT1) and hypoxia-inducible factors (HIF-1alpha and HIF-2alpha) [152]. Based on experimental studies, SGLT2i may activate all these pathways, and the interplay of all these mediators may stimulate autophagy. This lysosomal-mediated degradative pathway is responsible for the clearance of damaged organelles and, consequently, for the reduction of inflammasome activation and the mitigation of cardiomyocyte dysfunction and coronary microvascular injury [153,154].

## 4. Cardiovascular Benefits of SGLT2i: Clinical Outcomes and Impact on MACEs

The 2019 guidelines on diabetes, prediabetes and cardiovascular diseases of the European Society of Cardiology (ESC) and the European Association for the Study of Diabetes (EASD) recommend the use of SGLT2i in patients with T2DM and CVD or, at a very high/high cardiovascular (CV) risk, to reduce CV events (class I, level A) [155]. The long-established oral glucose-lowering drug impacts on cardiovascular outcomes have not been evaluated in large, randomized controlled trials. On the other hand, growing evidence from randomized trials and observational studies show that the treatment with SGLT2i reduces the risk of serious CV complications and death in patients at risk for major adverse cardiac events (MACE) [156]. Four large CV outcome studies have recently been completed: EMPA-REG OUTCOME, CANVAS, DECLARE-TIMI 58 and VERTIS-CV [110,113,157,158].

These studies showed a clear decrease in hospitalizations for HF in patients treated with SGLT2. This effect was observed in patients both with and without pre-existing HF and in those with and without pre-existing CVD. A reduction in the all-cause and CV mortality was also observed, particularly significant in the EMPA-REG and CANVAS studies but not in DECLARE-TIMI 58.

A recent meta-analysis supported the conclusion that SGLT2i are effective in reducing the risk of HF hospitalization in a large population of individuals with diabetes, regardless of a prior history of CVD. However, the reduction in the incidence of MACE is moderate and limited to patients with established atherosclerotic CVD [159].An overview of the main trials and related findings is presented in Table 1.

The Empagliflozin, Cardiovascular Outcomes, and Mortality in Type 2 Diabetes (EMPA-REG OUTCOME) study examined the efficacy and safety of empagliflozin in reducing the mortality and CV morbidity in 7020 subjects with diabetes at high risk for CV events [113]. The patients were randomly assigned either to Empagliflozin (10 or 25 mg) once-daily in addition to the standard care or to the placebo (median follow-up period of 3.1 years). Compared to the placebo, Empagliflozin showed a significantly lower risk of death from CV causes (−38%), death from any cause (−32%) and hospitalization for HF (−35%). No significant finding instead emerged as for MI/stroke incidence [113]. The results overlapped for both dosages of Empagliflozin. These findings are relevant considering the demonstrated superiority of Empagliflozin over the standard care. Most patients were already receiving antihypertensive drugs (94%) and statins (77%). Though the treatment proved to reduce the primary endpoint (CV mortality, nonfatal MI or nonfatal stroke), it must be highlighted that statistically significant reductions in the primary outcome were observed only in specific subgroups (e.g., age ≥ 65, A1C < 8.5%, Asian race and BMI < 30). Particularly, an important geographic variation in CV deaths was observed. Europe and North America (representing more than half of the study population) showed no significant reduction in CV deaths, while Asia and Latin America showed a significant reduction. Moreover, Empagliflozin was revealed as effective in preventing CV death, but no effect was observed on the specific components of CV disease, such as stroke and MI.

The CANagliflozin cardioVascular Assessment Study (CANVAS) program integrated data from two studies (CANVAS and CANVAS-renal), involving 10,142 participants with diabetes and a high CV risk. In each study, patients were randomized either to Canagliflozin (100 or 300 mg) or the placebo (mean follow-up: 188.2 weeks). Canagliflozin demonstrated superiority over the placebo with regards to the primary outcome. However, none of the individual components of MACEs, nor all-cause mortality, disclosed a significant reduction, whilst, similar to Empagliflozin, a benefit emerged in the reduction of HF hospitalization rates (RR 0.67; 95% CI 0.52–0.879) [157]. The lack of homogeneity in the baseline characteristics of enrolled patients in EMPA-REG OUTCOME and those in the CANVAS program might explain the differences observed in the CV outcomes. In fact, while just two-thirds of patients in the CANVAS trial were affected by a CV disease, EMPA-REG enrolled 99.5% of subjects with prior CV events. It can be speculated that the higher baseline risk allowed a better CV protection, as shown in the EMPA-REG trial. Indeed, if we considered only the CANVAS subjects with prior CV events, they had an 18% reduction in CV deaths [157].

The Dapagliflozin Effect on Cardiovascular Events–Thrombolysis in Myocardial Infarction 58 (DECLARE–TIMI 58) study is a randomized, phase III, double-blind, multicenter, placebo-controlled trial that assessed the daily treatment with Dapagliflozin 10 mg in patients with T2DM and established atherosclerotic CV disease with multiple risk factors [110]. The study failed to achieve the primary endpoint. Indeed, Dapagliflozin is noninferior for reducing MACEs in patients with DM2 and a high CV risk. In the Dapagliflozin group, a lower incidence of the composite outcome “CV death or HF hospitalization” was observed, as compared with the placebo (4.9% vs. 5.8%; RR 0.83; CI 95% 0.73–0.95). Of note, the lower incidence of the composite outcome in the Dapagliflozin group was due to a lower percentage of HF hospitalizations (RR 0.73; 95% CI 0.61–0.88). In fact, the CV mortality was similar in the two treatment groups (RR 0.98; 95% CI 0.82–1.07). Evidence for the protective effects against MI, stroke and CV death was limited to patients with pre-existing overt atherosclerotic disease.

The Evaluation of Ertugliflozin Efficacy and Safety Cardiovascular Outcomes (VERTIS CV) is a randomized, placebo-controlled, double-blind study in which 8246 patients with T2DM and high CV risk were recruited and randomized in a 1:1:1 ratio to receive Ertugliflozin (5/15 mg) or a placebo, in addition to standard therapy (mean follow-up: 3.5 years) [160]. The primary outcome (composite of CV death, nonfatal MI and nonfatal stroke) occurred in 11.9% of patients in both Ertugliflozin groups and 11.9% of patients in the placebo group (HR 0.97, CI 95% 0.85–1.11; *p* < 0.001 for noninferiority). The secondary outcome (composite outcome cv. death and hospitalization for HF), indeed, occurred in 8.1% of subjects in both Ertugliflozin groups and 9.1% of patients in the placebo group (HR 0.88, CI 95% 0.75–1.03; *p* = 0.11 for superiority). Ertugliflozin, in addition to the standard therapy, proved noninferior to the placebo in terms of the incidence of MACEs in a population of patients, with T2DM at a very high CV risk. Finally, the incidence of the composite outcome “CV death and hospitalization for HF” and the composite renal outcome did not differ between the study groups. Ertugliflozin failed to match its rivals in providing benefits over the placebo for a composite of CV death or hospitalization for heart failure, CV death and a composite of renal death and decline. These different outcomes did not find a clear explanation when compared with those observed in previous trials on SGLT2i. A hypothesis postulated by the authors could stand in the increasing aggressiveness, in most recent years, of secondary CV prevention therapies. Of note, the VERTIS-CV trial had the higher proportion of patients with HF (~24%) as compared to other major CV outcome trials (~10–15%). Furthermore, there are several differences in the CV risk among these trials. The EMPA-REG and VERTIS-CV trials enrolled patients with established atherosclerotic CV disease, while CANVAS and DECLARE-TIMI included patients with either established atherosclerotic CV disease or multiple CV risk factors, which could have affected the CV event incidence between trials. Besides, the more widespread use of other hypoglycemic drugs with proven cardiorenal benefits would have rendered it more difficult to reach a significance, even in the presence of a favorable trend, although it cannot be excluded that small differences between drugs in the class result in different outcomes. However, hospitalization for HF was absolutely consistent with what was observed in previous studies with other SGLT2i, thus confirming once again the efficacy of this class of drugs on this side [159].

The encouraging data from SGLT2i trials on MACEs and HF have led to several sub-analyses or new studies focused on the class effect on worsening HF and HF hospitalizations. A recent sub-analysis of VERTIS-CV aimed to evaluate the effect of Ertugliflozin on hospitalization for HF [160]. Ertugliflozin did not significantly reduce the composite “first HF hospitalization/CV death” (HR, 0.88 (95% CI, 0.75–1.03)), whilst a reduced risk was observed as for first HF hospitalization (HR, 0.70 (95% CI, 0.54–0.90); *p* = 0.006) [161].

The CANVAS sub-analysis, indeed, showed that CV death or HF hospitalization was reduced in patients treated with Canagliflozin as compared to the placebo (16.3 vs. 20.8 per 1000 patient/year; HR 0.78; 95% CI 0.67–0.91). Similar findings also emerged as for fatal/hospitalized HF (HR 0.70; 95% CI 0.55–0.89) and hospitalized HF alone (HR 0.67; 95% CI 0.52–0.87) [161].

The DAPA-HF (Dapagliflozin And Prevention of Adverse-outcomes in Heart Failure) study was instead the first international, multicenter, parallel-group, randomized, double-blind clinical trial on a SGLT2i designed to assess the effect of Dapagliflozin 10 mg (once-daily, in addition to the standard care) vs. a placebo in 4744 patients with HFrEF (left ventricle ejection fraction ≤40%) and NYHA classes II–IV, both with and without T2DM [162]. After a median follow-up of 18 months, the primary endpoint (composite outcome of worsening HF) occurred in 386 of 2373 patients (16.3%) in the Dapagliflozin group and in 502 of 2371 patients (21.2%) in the placebo group (HR 0.74, 95% CI 0.65–0.85, *p* < 0.001). A first event of worsening HF occurred in 237 patients (10.0%) in the Dapagliflozin group and in 326 patients (13.7%) in the placebo group (HR = 0.70, CI 95% = 0.59–0.83). Of note, the overall effect of Dapagliflozin on the primary endpoint remained significant even in patients without diabetes [163]. Differential benefits were found according to the NYHA symptom class. The main benefit was observed among patients with NYHA class II symptoms. The large use of mineralocorticoid receptor antagonists and other drugs against HF, resulting in benefits among the subgroups, rendered the results contemporary and relevant. However, the author-noted limitations include the low prevalence of sacubitril–valsartan use (11%).

The EMPEROR-Reduced (EMPagliflozin outcomE tRial in Patients With chrOnic heaRt Failure With Reduced Ejection Fraction) trial is a phase III, multicenter, randomized, placebo-controlled, double-blind study on 3730 patients with HFrEF [163]. The authors assessed the efficacy of Empagliflozin (10 mg as single daily dose) the vs. placebo in reducing CV mortality and HF hospitalizations in patients with HFrEF, both with or without diabetes and a history of HF (NYHA classes II-IV) and left ventricular ejection fraction ≤40%. After a median follow-up of 16 months, the incidence of the primary endpoint (death from CV causes or hospitalization for HF) was 19.4% in the Empagliflozin group vs. 24.7% in the placebo group (HR 0.75, 95% CI 0.65–0.86; *p* < 0.001). This positive finding was mainly ascribed to the reduction in HF hospitalizations (HR 0.69, 95% CI 0.59–0.81), whereas the reduction in CV death did not reach statistical significance (HR 0.92, CI 95% 0.75–1.12). Enrolled patients were largely treated with well-proven efficacy drugs in HFrEF management. In the Empagliflozin arm, 94.7% received beta-blockers, 70.5% angiotensin-converting enzyme inhibitors or angiotensin II receptor antagonists, 70.1% mineralocorticoid receptor antagonist and 18.3% sacubitril–valsartan, with no significant differences as compared to the placebo group. The reduction in the primary outcome was independent on the concomitant treatment, suggesting both a protective effect of Empagliflozin “on top” of HF therapy, as well as the safety and feasibility of such a coadministration. The results of the EMPEROR-Reduced trial are consistent with those of the DAPA-HF trial and support the use of SGLT2i in patients with chronic HFrEF, regardless of the history of diabetes [164,165]. These two trials share similarities and differences, both in the study design and results. Patients in EMPEROR-Reduced, as compared to DAPA-HF, had a lower left ventricle ejection fraction (27% vs. 31%), higher baseline NT-proBNP values (~1900 vs. 1430 pg/mL) and worse renal function (~61 vs. ~66 mL/min/1.73 m^2^). These characteristics might explain the earlier achievement of the prespecified number of events and the shorter follow-up. Other major baseline characteristics were similar in the two trials; the prevalence of patients with and without diabetes was around the 50%, and the medical therapy of HFrEF was optimized.

SGLT2i have become a new class of drugs for chronic HFrEF, regardless of the history of diabetes, with a new and different mechanism of action from that of the neurohormonal antagonists on which HFrEF therapy has been previously based. Moreover, recent trials such as DAPA-HF and EMPEROR-Reduced enrolled more than half of nondiabetic patients, thus confirming the use of SGLT2i as drugs for HF treatment more than for T2DM. Based on the evidence, the Food and Drug Administration in 2020 approved Dapagliflozin as the first SGLT2 inhibitor available to treat patients with HFrEF or a left ventricular ejection fraction less than 40%, regardless of the presence of diabetes. Likewise, Dapagliflozin has been approved in the European Union for the treatment of symptomatic chronic HFrEF in patients both with and without T2DM. Moreover, the recent findings from EMPA-TROPISM (Are the “Cardiac Benefits” of Empagliflozin Independent of Its Hypoglycemic Activity?) trial confirm efficacy in nondiabetic patients. The treatment with Empagliflozin, as compared to the placebo, in nondiabetic HFrEF patients significantly improves left ventricle volumes, mass, systolic function, functional capacity and quality of life [166].

Due to these reasons, SGLT2i would change our clinical practice. However, their efficacy in other settings, including HF with preserved ejection fraction and acute HF, still remains to be investigated. Hypothetically, to date, no drug therapy has demonstrated a CV outcome benefit in patients with HFpEF, as demonstrated by SGLT2i. These latter exert a favorable effect on the cornerstone of HFpEF treatment: left ventricle diastolic function improvement and left ventricle filling pressure reduction. Based on these considerations and on the clinical unmet needs, a plethora of trials on the use of SGLT2i in HFpEF patients are ongoing, aiming to establish their roles in this setting. In the PRESERVED-HF study (Dapagliflozin in PRESERVED Ejection Fraction Heart Failure), the investigators aimed to assess the impact of Dapagliflozin, as compared to the placebo, on HF, disease-specific biomarkers, symptoms, health status and quality of life in patients with HFpEF (NCT03030235). The ongoing DELIVER (Dapagliflozin Evaluation to Improve the LIVEs of Patients With PReserved Ejection Fraction Heart Failure) study is a phase III, multicenter, event-driven, randomized, double-blind, parallel-group, placebo-controlled study in patients with HFpEF. The DELIVER aims to assess the effects of Dapagliflozin, once-daily in addition to the standard care, on the reduction of the composite of CV death or HF events (NCT03619213) in patients with HFpEF, either with or without diabetes, with CV risk factors and/or established CVD. Likewise, the DETERMINE-Preserved (Dapagliflozin Effect on Exercise Capacity Using a 6-min Walk Test in Patients With Heart Failure With Preserved Ejection Fraction) study aims to evaluate the effects of Dapagliflozin in patients with a preserved ejection fraction (NCT03877224). The EMPERIAL (Effect of EMPagliflozin on ExeRcise ability and heart failure symptoms In patients with chronic heArt faiLure)-Preserved trial (NCT03448406) is a phase III, randomized, double-blind, placebo-controlled aimed to investigate the effects of Empagliflozin on the exercise capacity and patient-reported outcomes in patients with HFpEF. EMPEROR-Preserved (NCT03057951) is a phase III randomized, double-blind, parallel-group, placebo-controlled trial on 5988 symptomatic HFpEF patients both with and without T2DM. The investigators aimed to assess whether Empagliflozin lowers the risk of hospitalization for heart failure and improves survival.

In the latter setting, particularly, recent data with Sotagliflozin [167] and preliminary data with Empagliflozin have been reported [168], and further results are expected soon from the ongoing trials [169].

Of note, the antiproteinuric effect of SGLT2i is extremely evident in all RCTs. This mechanism could justify the reduction of both CV and renal risk in T2DM. In fact, the impact of the modification of proteinuria on the cardiorenal risk has been documented by recent real-life studies [170].

## 5. Conclusions

Several potential mechanisms may explain the ability of SGLT2i to decrease the CV risk and, particularly, hospitalization for HF in patients with or without T2DM. Their effect seems to go beyond the simple hyperglycemic control, since it was not observed in other antidiabetic drugs with more significant glucose-lowering effects. The beneficial effects seem attributable to the significant reduction of the intracellular sodium levels, well-known to exert a cardioprotective role in the prevention of oxidative stress and consequent cardiomyocyte death. From a molecular perspective, patients’ exposure to gliflozin treatment mimics nutrient and oxygen deprivation, with consequent autophagy stimulation. This allows to maintain cellular homeostasis through different degradative pathways. Thus, since their introduction in the clinical practice, the hypothesis on SGLT2i mechanisms of action has changed: from simple glycosuric drugs with consequent glucose lowering, erythropoiesis enhancing and ketogenesis stimulating to intracellular sodium-lowering molecules. These mechanisms of action result in a significant reduction in CV events.

Based on clinical evidence, the use of SGLT2i seems crucial in clinical practice, particularly in the treatment of HF patients. An early use of SGLT2i could exert a considerable impact on the prognosis in a real-life scenario. Therefore, in our opinion, SGLT2i should be introduced in the first step of the treatment, regardless of concomitant medications. In fact, in several trials, they have been shown as effective irrespective of the baseline medication and of the current step-by-step HF treatment, which often produces time dilation, thus affecting the prognosis. In the near future, we imagine that SGLT2i may be used as a first-line therapy, potentially representing a cornerstone to build the HF therapy.

## Figures and Tables

**Table 1:** BMI: Body Mass Index. CVD: Cardiovascular Disease. eGFR: Estimated Glomerular Filtration Rate. HF: Heart Failure. HHF: Hospitalization for Heart Failure. HFrEF: Heart Failure with reduced Ejection Fraction. HFpEF: Heart Failure with preserved Ejection Fraction. MACE: Major Adverse Cardiovascular Events. NYHA: New York Heart Association. T2DM: Type 2 Diabetes Mellitus. MI: myocardial infarction. † Two or more of the following risk factors for cardiovascular disease: duration of diabetes of at least 10 years, systolic blood pressure higher than 140 mmHg while they were receiving one or more antihypertensive agent, current smoking, microalbuminuria or macroalbuminuria or high-density lipoprotein (HDL) cholesterol level of less than 1 mmol per liter (38.7 mg per deciliter). * Defined as clinically evident ischemic heart disease, ischemic cerebrovascular disease or peripheral artery disease. ** Hypertension, dyslipidemia (defined as a low-density lipoprotein cholesterol level >130 mg per deciliter (3.36 mmol per liter) or the use of lipid-lowering therapies) or the use of tobacco. ‡ LVEF 31–35% > 1000, resp. 2000 in patient with atrial fibrillation and LVEF 36–40% > 2500, resp. 5000 in pt with atrial fibrillation.

Trial (SGLT2 Inhibitor)	Patients n.	Inclusion Criteria	Study Design	Mean HbA1c—% [mmol/mol]	Mean BMI (kg/m^2^)	Heart Failure	Mean eGFR (mL/min/ 1.73 m^2^)	Endpoints	Follow-Up	Outcomes
*EMPA-REG* *(Empagliflozin)*	7020	- T2DM - Age ≥ 18 years - BMI ≤ 45 kg/m^2^ - eGFR ≥ 30 mL/min/1.73 m^2^ - CVD	Empagliflozin 10 mg daily (n = 2345) Empagliflozin 25 mg daily (n = 2342) Placebo (n = 2333)	8.1 [65]	30.6	HF (unspecified): 706 (10.1%)	74.0	Primary outcome: CV mortality, nonfatal MI, or nonfatal stroke Key secondary outcome was a composite of the primary outcome plus hospitalization for unstable angina.	Median follow-up: 3.1 years	CV mortality, nonfatal MI, or nonfatal stroke 10.5% vs 12.1% 0.86 (0.74–0.99) Composite of the primary outcome plus hospitalization for unstable angina 0.89 (0.78–1.01) HHF 0.65 (0.50–0.85)
*CANVAS* *(Canagliflozin)*	10,142 (4330 in CANVAS and 5812 in CANVAS-R)	- T2DM with HbA1c ≥ 7.0% to ≤10.5% at screening - Not currently on antihyperglycemic agent therapy or on antihyperglycemic monotherapy or combination therapy -History or high risk of CV disease †.	Canagliflozin 100 mg vs. 300 mg daily vs. Placebo - Canagliflozin 100 mg then 300 mg daily vs. Placebo	8.2 [66]	32.0	HF(unspecified): 1461 (14.4%)	76.5	Primary outcome: composite of death from CV causes, nonfatal MI, or nonfatal stroke. Secondary outcomes: death from any cause, death from CV causes, progression of albuminuria, and the composite of death from CV causes and HHF.	Median follow-up: 126 weeks; mean follow-up: 188 weeks	Composite of death from CV causes, nonfatal MI, or nonfatal stroke 0.86 (0.75–0.97) Death from any cause 0.87 (0.74–1.01) Composite of death from CV causes and HHF 0.78 (0.67–0.91) HHF 0.67 (0.52–0.87)
*DECLARE- TIMI* *(Dapagliflozin)*	17,160	- ≥ 40 years of age - Diagnosed with T2DM - High risk for CV event * OR No known CVD AND at least 2 factors in addition to T2DM **	Dapagliflozin 10 mg daily (n = 8852) Matching 1:1 Placebo (n = 8578)	8.3 [67]	32.1	HFrEF: 671 (3.9%) HFpEF: 1316 (7.7%)	85.2	Primary safety outcome: MACE (CV death, MI, or ischemic stroke). Two primary efficacy outcomes: MACE and a composite of CV death or HHF.	Median follow-up: 4.2 years	MACE 8.8% vs 9.4% 0.93 (0.84−1.03) CV death or HHF 4.9% vs 5.8% 0.83 (0.73−0.95) HHF 0.73 (0.61−0.88)
*VERTIS-CV* *(Ertugliflozin)*	8246	≥ 40 years of age - Diagnosed with T2DM (with a glycated hemoglobin level of 7.0 to 10.5%) - established atherosclerotic CV disease	Ertugliflozin 5 mg or 15 mg of or matching placebo once daily	8.2 [66]	32.0	HF (unspecified): 23.4%	76.0	Primary outcome: composite of death from CV causes, nonfatal MI, or nonfatal stroke. Key secondary outcomes: a composite of death from CV causes or HHF; death from CV causes	Mean follow-up: 3.5 years	Composite of death from CV causes, nonfatal MI, or nonfatal stroke 0.97 (0.85–1.11) Composite of death from CV causes or HHF 0.88 (0.75–1.03) Death from CV causes 0.92 (0.77–1.11) HHF 0.70 (0.54–0.90)
*DAPA-HF* *(Dapagliflozin)*	4744	- Age ≥ 18 years - EF ≤ 40% - NYHA Class II, III, or IV symptoms - Plasma NT-proBNP level of: ≥ 600pg/mL OR ≥ 400pg/mL if HHF within the past 12 months OR ≥ 900 pg/mL if patient had AF/flutter on baseline ECG.	Dapagliflozin 10 mg once daily or matching placebo	T2DM—41.8% Non-T2DM— 58.2%	28.2	HFrEF: 4744 (100%)	65.8	Primary outcome: composite of worsening HF or death from CV causes. Key secondary outcome: composite of HHF or CV death	Median follow-up: 18.2 months	Composite of worsening HF or death from CV causes 0.74 (0.65 to 0.85) Composite of HHF or CV death 0.75 (0.65 to 0.85) HHF 0.70 (0.59 to 0.83)
*EMPEROR-reduced* *(Empagliflozin)*	3730	- NYHA II-IV - Age > 18 yrs - HFrEF AND NT-proBNP > 600 pg/mL (1200 in pt with AF) - If LVEF 31-40% + recent HHF (in past 12 months) OR significantly elevated NT-proBNP ‡	Empagliflozin 10 mg daily or placebo	T2DM—49.8% Non-T2DM— 50.2%	27.9	HFrEF: 3730 (100%)	62.0	Primary outcome: composite of CV death or HHF Secondary outcome: HHF	Median follow-up: 16 months	Composite of CV death or HHF 0.75 (0.65 to 0.86) HHF 0.69 (0.59 to 0.81)

## Data Availability

Not available.
